# The incidence of training responsiveness to cardiorespiratory fitness and cardiometabolic measurements following individualized and standardized exercise prescription: study protocol for a randomized controlled trial

**DOI:** 10.1186/s13063-016-1735-0

**Published:** 2016-12-19

**Authors:** Ryan M. Weatherwax, Nigel K. Harris, Andrew E. Kilding, Lance C. Dalleck

**Affiliations:** 1 Auckland University of Technology, Human Potential Centre, Auckland, New Zealand; 2 Auckland University of Technology, Sports Performance Research Institute New Zealand, Auckland, New Zealand; 3 Department of Recreation, Exercise, and Sport Science, Western State Colorado University, Gunnison, CO USA

**Keywords:** Responder, Non-responder, Exercise prescription, Ventilatory threshold, Cardiovascular disease, Primary prevention

## Abstract

**Background:**

There is individual variability to cardiorespiratory fitness (CRF) training, but the underlying cause is not well understood. Traditionally, a standardized approach to exercise prescription has utilized relative percentages of maximal heart rate, heart rate reserve (HRR), maximal oxygen uptake (VO_2_max), or VO_2_ reserve to establish exercise intensity. However, this model fails to take into consideration individual metabolic responses to exercise and may attribute to the variability in training responses. It has been proposed that an individualized approach would take into consideration metabolic responses to exercises to increase responsiveness to training.

**Methods:**

In this randomized control trial, participants will undergo a 12-week exercise intervention using individualized (ventilatory thresholds) and standardized (HRR) methods to prescribe CRF training intensity. Following the intervention, participants will be categorized as responders or non-responders based on changes in maximal aerobic abilities. Participants who are non-responders will complete a second 12-week intervention in a crossover design to determine whether they can become responders with a differing exercise prescription. There are four main research outcomes: (1) determine the cohort-specific technical error to use in the categorization of response rate; (2) determine if an individualized intensity prescription is superior to a standard approach in regards to VO_2_max and cardiometabolic risk factors; (3) investigate the time course changes throughout 12 weeks of CRF training between the two intervention groups; and (4) determine if non-responders can become responders if the exercise prescription is modified.

**Discussion:**

The findings from this research will provide evidence on the effectiveness of individualized exercise prescription related to training responsiveness of VO_2_max and cardiometabolic risk factors compared to a standardized approach and further our understanding of individual exercise responses. If the individualized approach proposed is deemed effective, it may change the way exercise specialists prescribe exercise intensity to enhance training responsiveness.

**Trial registration:**

ClinicalTrials.gov, NCT02868710. Registered on 15 August 2016.

**Electronic supplementary material:**

The online version of this article (doi:10.1186/s13063-016-1735-0) contains supplementary material, which is available to authorized users.

## Background

Heterogeneity in the response to exercise training first received attention in the 1980s [1] with a series of standardized studies investigating trainability of sedentary adults. Among these studies was an investigation into responses of maximal aerobic power in which it was reported that interindividual differences ranged from 5% to 88% [2]. Even though these original findings were reported over 30 years ago, substantial individual variability in response to prescribed exercise regimes remains a poorly understood phenomena. Nonetheless, it has been purported that a more individualized approach to the exercise prescription may enhance training efficacy and limit training unresponsiveness. For instance, it has been acknowledged as far back as the late 1970s that utilizing a relative percent method (i.e., % heart rate reserve [HRR]) to establish exercise intensity fails to account for individual metabolic responses to exercise [3]. Nevertheless, the relative percent concept remains the gold standard recommendation for exercise intensity [4]. It is both plausible and practical to think that an intensity set based on an individual’s threshold measurement (i.e. ventilatory threshold) will not only encourage more positive physiological adaptations, but may account for some of the variability in training responsiveness by taking into consideration individual metabolic differences. Based on an extensive search of the literature, to our knowledge, there is only one investigation that set out to determine the incidence of response based on exercise prescription using standard methods (%HRR) compared to individualized methods (threshold based) in which they found 100% of the individualized group responded in a positive manner [5]. However, this investigation had several limitations including a modest intervention duration, only reported maximal oxygen uptake (VO_2_max) changes, and sourced measurements for biological variability to use as criteria for response rate rather than testing for biological variability within the laboratory where data were collected.

A notable factor that confounds current understanding of training response variability is the absence of a set definition in the literature of how to interpret a response (i.e. what classifies someone as a responder or non-responder). Indeed, criteria to determine incidence of response for changes in VO_2_max have included classifying a fixed proportion of the lowest training response [6], absolute changes in pre- to post-intervention values [7, 8], and a change of more than one standard deviation [9]. More recently, it has been proposed that technical error (TE), the combination of day-to-day biological variability and measurement error, should be applied to categorize response rate [10]. If these values are considered for each research cohort to report incidence of response, there would be greater consistency of reporting results within the literature to provide further insight on individual variability. Moreover, interpretation of the individual variability in training responsiveness is limited due in part to the standard practice of past studies only reporting group means and standard deviations. With reporting of only the mean and standard deviation, results of the intervention may not be applicable to all since there is a lack of understanding related to the individual variability of the investigation.

This trial will be the first investigation to address the incidence of response of VO_2_max and cardiometabolic risk factors following individualized and standardized cardiorespiratory fitness (CRF) training using a specific cohort-calculated TE as the criteria for response. Similarly, this will be the first study to evaluate the efficacy of modifying the CRF training intensity prescription for non-responsive participants (based on changes of VO_2_max) to investigate subsequent training responsiveness.

### Research aims

The objective of this research is to determine the incidence of response to VO_2_max after implementation of a standardized (%HRR) and individualized (based on first ventilatory threshold [VT1] and second ventilatory threshold [VT2]) approach to exercise prescription in a community wellness program for 12 weeks. The primary measurement outcome will be VO_2_max with secondary outcomes of total cholesterol (TC), high-density lipoprotein (HDL), low-density lipoprotein (LDL), triglycerides, blood glucose, and resting heart rate (HR) and blood pressure (BP). Thus, the main research aim is to determine whether an individualized exercise prescription decreases the incidence of non-response to CRF and cardiometabolic measurements compared to the standardized approach. Similarly, another primary research aim is to determine if changing the prescription of CRF intensity for a subsequent 12-week intervention elicits more responsiveness in previously categorized non-responders (based on VO_2_max responses). A key secondary aim of this research is to establish whether there are differences in the time course changes of VO_2_max and cardiometabolic risk factors between the experimental groups every fourth week during the 12-week intervention.

## Methods/Design

**Fig. 1:**
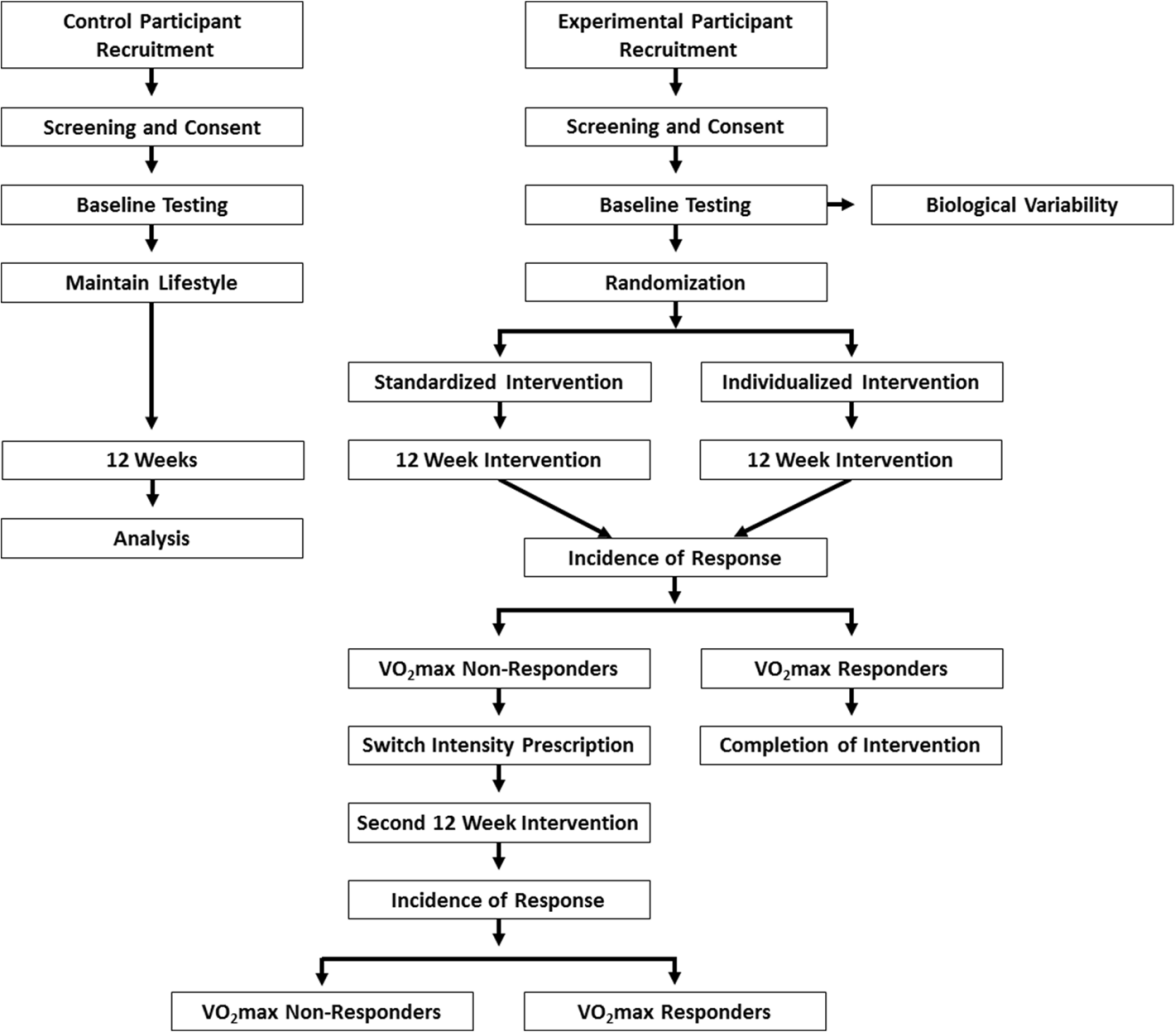
Schematic illustration of the research design

The Standard Protocol Items: Recommendations for Interventional Trials (SPIRIT) guidelines [11] have been taken into consideration for the planning of this trial (Additional file 1). The overall study (Fig. 1) is a randomized control trial with participants completing a CRF training study 3 days a week for a duration of 12 weeks using a standardized (%HRR) and an individualized (based on VT1 and VT2) approach (see Additional file 2: overall SPIRIT study schedule or Tables 1, 2 and 3 for SPIRIT schedule design). If participants do not have a favorable outcome after 12 weeks (i.e. non-responders), they will complete a second 12-week intervention with a crossover design of the other exercise prescription. The protocol has been approved by the Auckland University of Technology Ethics Committee (16/264) and the Human Research Committee of the Institutional Review Board at Western State Colorado University (HRC2016-01-90R6) with data collection occurring only at Western State Colorado University.

**Table 1:** SPIRIT study calendar for the experimental group for the first 12-week intervention

			Exercise intervention				
Point of time:	Pre-intervention	Baseline	Time 0	Week 4	Week 8	Week 12	12-week analysis
Enrollment							
Recruitment	x						
Medical history	x						
Inclusion criteria	x						
Randomization			x				
Testing							
Height		x		x	x	x	
Weight		x		x	x	x	
Resting heart rate		x		x	x	x	
Resting blood pressure		x		x	x	x	
Waist circumference		x		x	x	x	
Low-density lipoprotein		x		x	x	x	
High-density lipoprotein		x		x	x	x	
Triglycerides		x		x	x	x	
Blood glucose		x		x	x	x	
Maximal exercise test		x		x	x	x	
Verification test		x		x	x	x	
3-day nutrition recall		x				x	
Analysis							
Biological variability			x				
Time course changes							x
Incidence of response							x

*SPIRIT* Standard Protocol Items: Recommendations for Interventional Trials

**Table 2:** SPIRIT study calendar for the control group for the first 12-week intervention

Point of time:	Pre-intervention	Baseline	Time 0	Week 4	Week 8	Week 12	12-week analysis
Enrollment							
Recruitment	x						
Medical history	x						
Inclusion criteria	x						
Testing							
Height		x		x	x	x	
Weight		x		x	x	x	
Resting heart rate		x		x	x	x	
Resting blood pressure		x		x	x	x	
Waist circumference		x		x	x	x	
Low-density lipoprotein		x		x	x	x	
High-density lipoprotein		x		x	x	x	
Triglycerides		x		x	x	x	
Blood glucose		x		x	x	x	
Maximal exercise test		x		x	x	x	
Verification test		x		x	x	x	
3-day dietary recall		x				x	
IPAQ		x		x	x	x	
Analysis							
12-week changes							x

*SPIRIT* Standard Protocol Items: Recommendations for Interventional Trials, *IPAQ* International Physical Activity Questionnaire

**Table 3:** SPIRIT study calendar (second 12 weeks) for VO_2_max non-responders after the first 12-week intervention

			Exercise intervention				
Point of time:	Pre-intervention	Baseline	Time 0	Week 4	Week 8	Week 12	12-week analysis
Pre-intervention							
Switch of experimental group	x						
Testing							
Height		x		x	x	x	
Weight		x		x	x	x	
Resting heart rate		x		x	x	x	
Resting blood pressure		x		x	x	x	
Waist circumference		x		x	x	x	
Low-density lipoprotein		x					
High-density lipoprotein		x					
Triglycerides		x					
Blood glucose		x					
Maximal exercise test		x		x	x	x	
Verification test		x		x	x	x	
3-day nutrition recall		x				x	
Analysis							
Incidence of response							x

*SPIRIT* Standard Protocol Items: Recommendations for Interventional Trials, *VO2max* maximal oxygen uptake

### Sample – experimental groups

For the experimental groups, participants will be recruited from a community-based wellness program serving the local area. The wellness program participants are either referred by local medical professionals or seek entrance into the program from peer referrals. In order for participants to be included in the study, they must meet the following inclusion criteria:

- 30 to 75 years of age
- Considered low to moderate risk for cardiovascular disease [4]
- Currently sedentary (participating in less than 30 minutes of moderate intensity physical activity on at least 3 days a week)
- Resided at an altitude near 2300 m for at least the last 6 months

Participants will be excluded from the study if they have any signs, symptoms, or diagnosed cardiovascular, pulmonary, or metabolic disease. During the trial, participants will be asked to maintain their normal lifestyle to ensure any adaptations were due to the intervention.

### Sample – control group

Due to moral and ethical issues related to excluding participants from an exercise intervention to improve health, a control group will be recruited as a separate cohort. The control group participants must meet the same inclusion criteria and will not be allowed in the study if they meet the exclusion criteria, as previously mentioned.

### Sample size calculation

Sample size was projected with change in VO_2_max as the main outcome variable. The means and standard deviations of a previous study [5] were examined and the effect size for this research study was calculated. Assuming a power of 0.80 was needed and the calculated effect size for change in VO_2_max was 0.30, it was determined that approximately 16 participants would be needed for each group [12]. It is assumed there would be an approximate 20% dropout rate, so the aim will be to achieve 20 participants per group.

### Intervention

#### Testing

Testing sessions for both the experimental and control groups will be conducted at baseline and every fourth week during the 12-week intervention period. Testing sessions every fourth week will help to establish the current physiological levels to develop the exercise prescription for the experimental groups.

Testing will be conducted in a university-based performance laboratory under the supervision of three exercise physiologists. Prior to completing the testing sessions, participants will be asked to refrain from food and drink (other than water) for 12 hours prior to the testing session, be well hydrated prior, avoid the use of alcohol, caffeine, and tobacco within 24 hours of testing, be well rested, avoid significant exertion or exercise the day of testing, and report any medication use prior to testing. Testing will occur as close to the same time of day as possible with the above directions prior to each testing session. Following the blood lipid profile testing (explained in detail later) and prior to the maximal exercise test, participants will be provided a small snack. The testing will be conducted as follows:

- Dietary analysis: participants will be instructed to not change their usual diets throughout the study and asked to complete a 3-day dietary intake recall including two weekdays and one weekend day to evaluate energy intake and the proportion of kilocalories from carbohydrates, protein, and fat.
- Anthropometric measurements: participants will be weighed on a calibrated, medical-grade scale to the nearest 0.01 kg and height will be measured using a stadiometer to the nearest 0.5 cm. Waist circumference will be measured by the narrowest horizontal circumference above the umbilicus and below the xiphoid process to the nearest 0.5 cm [4].
- Resting heart rate (RHR) and BP measurements: procedures for RHR and BP will follow standard guidelines [4]. In summary, participants will be required to sit for 5 minutes with sufficient back support, feet on the ground, and arms supported at heart level. Resting heart rate will be recorded by using a medical-grade pulse oximeter after the 5 minutes of seated rest. Blood pressure will be measured using a stethoscope and sphygmomanometer to determine left arm brachial artery BP on consecutive measure separated by 1 minute. The mean of the systolic and diastolic measures will be considered the resting BP.
- Fasting blood glucose and lipid measurements: all fasting lipid and blood glucose measurements will be analyzed using the Cholestech LDX system (Alere, Waltham, MA, USA), which has been shown to have excellent reproducibility [13, 14]. An optics check of the Cholestech LDX system will be completed at the beginning of each testing session. Participants will be asked to thoroughly wash hands with soap and rinse with warm water. The skin will then be wiped with an alcohol swab and allowed to dry. Using a lancet, the distal end of the third digit of the right hand will be punctured and a finger stick sample will be collected using a 40 μl capillary tube with blood flowing freely into the tube without milking the finger. The blood sample will then be extracted into a commercially available test cassette for analysis. Measurements of TC, HDL, LDL, triglycerides, and blood glucose will be obtained. Upon completion of the blood profile testing and data collection, blood samples will be disposed of based on standard biohazard procedures.
- VO_2_max and verification bout: participants will complete a modified Balke, pseudo-ramp graded exercise test (GXT) on a power treadmill. Participants will walk or jog at a self-selected pace with an increase in incline of 1% every minute until volitional fatigue. Heart rate and expired gas will be measured continuously using a heart rate monitor and a calibrated metabolic analyzer, respectively. Data will be analyzed following guidelines previously reported [5]. In summary, gas exchange data will be time averaged for every 15 seconds, VO_2_max will be determined by averaging the last two 15 second samples, and maximal HR will be the highest achieved HR during the GXT.

Since a verification procedure has been found to be effective in middle-aged and older adults to confirm VO_2_max [15], this procedure will be used to ensure participants have reached maximal capacity. The verification trial will be performed 20 minutes after the GXT as recommended elsewhere [16] and has been confirmed to be an effective procedure at altitude [17]. The verification bout will consist of a workload that is 105% of the maximal workload during the GXT (last fully completed stage) as this workload has been shown to be sufficient to elicit verification test durations of 2–3 minutes [15, 18] and will continue until volitional fatigue. Analysis of the verification bout will follow the same protocol as the GXT. ‘True’ VO_2_max will be considered to be attained if the GXT and verification bout are within ± 3% [15]. If participants are unable to reach VO_2_max, they will be asked to repeat the trial no sooner than 24 h later.

The control group will be asked to maintain their normal lifestyle activity habits. Therefore, in addition to the previously stated battery of testing, control participants will also complete the International Physical Activity Questionnaire (IPAQ) every fourth week at the testing session.

#### Biological variability and technical error

In order to establish criteria to categorize participants as responders or non-responders, the biological variability will be established to determine the TE. Therefore, from the pool of experimental research participants, 15 participants will be randomly selected based on when the referral or inquiry into the wellness program is received (i.e., participants 1, 3, 5, 7, etc. until 15 confirmed participants have been reached. If there are not 15 participants after the first round, then participants 2, 4, 6, etc. will be asked until the desired total of 15 participants is met). Participants will be asked to complete the baseline testing assessments twice within a 2-week period to determine the day-to-day biological variability. The biological variability will be combined with the typical error of the equipment utilized (sourced from the literature and company of the equipment) to determine the TE. More details related to the statistical approaches are located in the statistical analysis section.

#### Exercise intervention

After the completion of the baseline testing, participants will be randomly allocated to either the individualized or standardized arms at a 1:1 ratio using a computerized stratified minimization sequence. One of the primary investigators will have knowledge of the treatment groups to which participants have been allocated in order to interpret test results and prescribe target exercise intensities. However, this same investigator will not be involved in the implementation of the exercise training programs (to be completed by research assistants) in order to mitigate researcher bias. Participants will then be asked to come to the laboratory on Monday, Wednesday, and Friday to take part in the community wellness program and subsequent research. Upon arrival each day, participants will be asked to rest comfortably for 5 minutes in the seated position. Then, their resting BP and HR will be recorded. Following the resting measurements, participants will complete a 5-minute warm-up starting at a low and progressively increasing intensity until they are ready to begin their CRF exercise session. At this point, participants will be asked to stay within the designated HR (described in further detail below) outlined on their exercise log as determined based on their experimental group and week of experimental trial. At approximately 1/3 and 2/3 the total session time, an exercise physiologist or research assistant will record their current HR, rating of perceived exertion (scale 1–10), intensity of aerobic equipment, and any other pertinent notes. At the end of the CRF exercise session, the participant will be asked to complete a cooldown in which the exercise intensity is gradually reduced. While resistance training is not part of this proposed experiment, it is an integral part of the community wellness program and could be a confounding factor to the overall incidence of response. Therefore, all participants will be asked to complete the resistance training after the CRF training session is completed in order to have consistency among all participants. During the first 4 weeks, there will be no resistance training. During the next 4 weeks (week 4–8), there will be a learning and anatomical adaptation phase to resistance training in which proper technique and range of motion will be emphasized and participants will be acclimated to the resistance training machines. During the last 4 weeks (week 8–12) participants will complete one set of 8–12 repetitions on eight machine-based resistance training exercises and progress to two sets by the end of the 12th week [19].

#### Determination of workload

For the standardized group, the workload will be determined based on %HRR and completed based on the following calculation:

$$ \mathrm{H}\mathrm{R}\mathrm{R}=\left[\left(\mathrm{Maximal}\kern0.5em \mathrm{H}\mathrm{R}\hbox{-} \mathrm{Resting}\kern0.5em \mathrm{H}\mathrm{R}\right)\times \mathrm{Desired}\kern0.5em \mathrm{Percentage}\right]+\mathrm{Resting}\kern0.5em \mathrm{H}\mathrm{R} $$

For the individualized group, the workload will be determined based on ventilatory threshold values as previously described [5, 20] to determine VT1 and VT2. The criteria used for determining VT1 and VT2 will be a visual analysis of figures of time plotted against the relative respiratory variable – ventilatory equivalents of oxygen (VE/VO_2_) and ventilatory equivalents of carbon dioxide (VE/VCO_2_). Determination of VT1 will be an increase in VE/VO_2_ with no increase in VE/VCO_2_ and moving away from linearity of VE, whereas VT2 will be a simultaneous increase in both VE/VO_2_ and VE/VCO_2_. Calculations of HR values associated with ventilatory threshold (VT) values will be calculated prior to exercise sessions and with following HR ranges:

- Target HR > VT1 = HR range of 10 bpm below VT1 to the HR at VT1
- Target HR ≥ VT1 to < VT2 = HR range of 15 bpm above VT1 and below VT2
- Target HR ≥ VT2 = HR range of 10 bpm above VT2

**Fig. 2:**
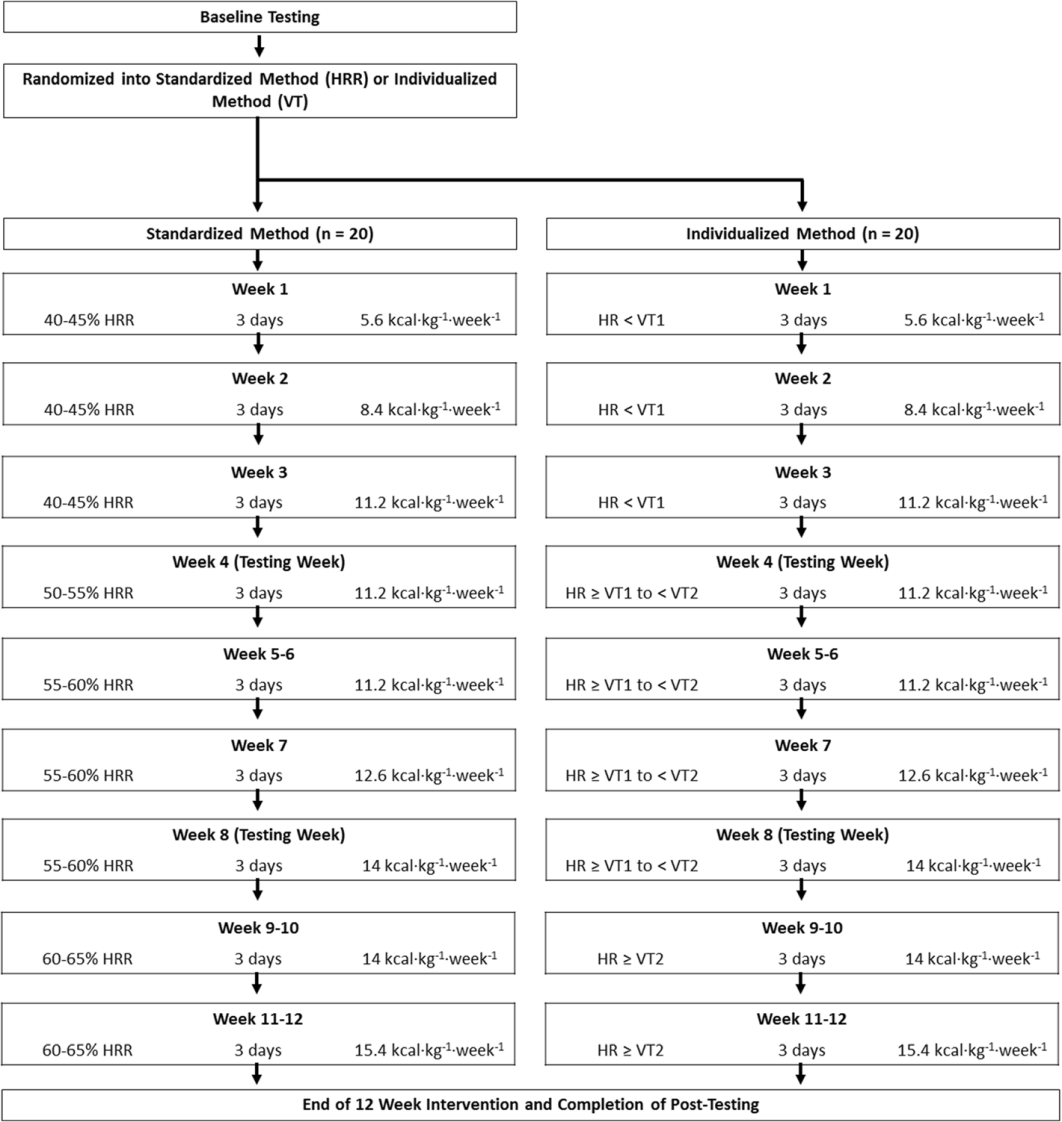
A detailed flow schematic of the exercise prescription for the experimental groups. *HRR* heart rate reserve, *kcal* kilocalories, *kg* kilograms, *VT1* first ventilatory threshold, *VT2* second ventilatory threshold

Exercise volume will be prescribed based on energy expenditure per kg of body weight a week (kcal · kg^−1^ · week^−1^) to implement an isocaloric exercise volume (i.e., in terms of kilocalories [kcal] per kg a week) across individuals and groups. Previous research has found that energy expenditure ranging from 4 kcal · kg^−1^ · week^−1^ [21] to 23 kcal · kg^−1^ · week^−1^ [22–25] have positive effects on CRF and cardiometabolic responses to exercise. Therefore, this study will utilize a similar 12-week exercise protocol as previously described [5], while implementing a standardized isocaloric volume (kcal · kg^−1^ · week^−1^) instead of a designated time for each exercise session. Exercise progression will follow standard guidelines that have been previously established [4]. Figure 2 illustrates the exercise progression following baseline testing through the 12-week intervention for both experimental groups while Tables 1 and 2 show the SPIRIT study schedule for experimental and control participants, respectively.

#### Time course changes over 12 weeks

Currently, there is no literature investigating the time course changes of VO_2_max and cardiometabolic risk factor values between a standardized and individualized CRF exercise program. Therefore, since testing will occur every fourth week, the time course changes over the first 12 weeks of the CRF training intervention will be highlighted and addressed. These data will help further analyze the incidence of response during 12 weeks of structured CRF training and gain insight into the time course changes associated with CRF training for each individual and based on the CRF training intensity (standardized or individualized).

#### Categorizing responders and non-responders

Following the 12-week intervention, the participants will be categorized as responders and non-responders (see statistical analysis section for further details) for each testing measurement. While each testing measurement will be evaluated, the overreaching categorization of responder and non-responder will be based on VO_2_max due to the profound health and performance implications of this measurement.

#### Modifying the prescription of non-responders

For the participants that are categorized as responders after the initial 12 weeks, they will be completed with the study. However, participants that were non-responders based on VO_2_max changes will be asked to complete a subsequent 12-week intervention. During this second 12-week intervention, participants will switch their exercise intensity prescription (i.e., if a participant in the standardized group was categorized as a non-responder, they will complete the second 12 weeks in the individualized group). The experimental design including the exercise prescription and testing (baseline and post-testing) will follow the same schedule and protocol. However, during week 4 and 8 the testing sessions will not include blood analysis. The SPIRIT study schedule for the second 12-week intervention can be seen in Table 3.

At the completion of the second 12-week intervention, the participants will again be categorized as responders or non-responders based on the new baseline and 12-week testing values. This crossover design intervention will help to gain insight on whether or not ‘non-responders’ can become responders if the intensity of the exercise prescription is modified.

### Data and confidentiality management

Electronic data will be coded, entered, and stored into a secure (password-protected) database on Western State Colorado University’s campus. All paper data, including consent forms, medical history documents, and daily exercise logs, will be stored in a secured locked cabinet in the Western State Colorado University Human Subject’s office. Only the primary investigators will have access to the data.

Due to the nature of the study, participants will be exercising in an environment with other members from the study. Therefore, anonymity of identity cannot be guarantee throughout the study. However, no research participant will be able to see or access any personal information – medical documents, exercise log, medications, etc. To ensure participant safety when exercising, researcher assistants delivering the exercise will be informed of relevant information that may influence how the participant responds to exercise. Any data collected and displayed in results of scientific manuscripts will be displayed in a way which does not disclose individual identity.

### Statistical analysis

All analyses will be performed using IBM SPSS Version 22.0 (Armonk, NY, USA) and GraphPad Prism 6.0 (GraphPad Software, San Diego, CA, USA).

#### Biological variability

Intraclass correlation (ICC) of variation, typical error and co-efficient of variation (CV) for VO_2_max, resting HR, resting BP, and fasting blood glucose, total cholesterol, HDL, and LDL will be calculated as described previously [26]. The CV will be combined with the measurement error of the testing to determine the TE. The TE will be used to categorize responders and non-responders. In summary, for all criteria tested the changes in pre- to post- intervention will be analyzed with responders having a change > TE and non-responders having a change that is ≤ TE.

#### Time course changes

Measures of centrality and spread will be presented as mean ± standard deviation (SD) and ranges will be reported for each measure. The precision of estimates will be reported as 90% confidence limits. Baseline-dependent variables will be compared using general linear model (GLM) ANOVA and Tukey post hoc tests, when applicable. Because baseline and every fourth week program data will be available after the completion of the first 12 weeks the effect of training on CRF (VO_2_max) and cardiometabolic measurements (TC, HDL, LDL, resting BP, and resting HR) will be determined using repeated-measures GLM-ANOVA with the exercise intensity (individualized or standardized) as the between subjects factor. Between group 12-week changes will be analyzed using GLM-ANOVA and Tukey post hoc tests, when appropriate. The assumption of normality will be tested by examining normal plots of the residuals in ANOVA models and will be regarded as normally distributed if Shapiro-Wilk tests are not significant [12]. Effect sizes will be calculated using means and pooled standard deviations. Method of data analysis will be analysis by treatment administered. Only participants who complete ≥ 70% exercise training sessions and strictly adhere to target exercise intensity will be included in the statistical analysis. The probability of making a type I error will be set at *p* ≤ 0.05 for all statistical analyses.

#### Incidence of response

Delta values (Δ) will be calculated (post-testing minus baseline value divided by baseline value) for percent change in relative VO_2_max, TC, HDL, LDL, resting BP and resting HR and participants will be categorized as: ‘1’ = responder (% Δ > TE) or ‘0’ = non-responder (% Δ ≤ TE). Chi-squared (*χ* ^2^) tests will be subsequently used to analyze the point prevalence of responders and non-responders to exercise training separated by exercise intensity group (individualized and standardized) between baseline and the end of the end the first 12-week intervention. Only participants who complete ≥ 70% exercise training sessions and strictly adhere to target exercise intensity will be included in the statistical analysis. The probability of making a type I error will be set at *p* ≤ 0.05 for all statistical analyses.

In order to make inferences about the true values (population values) of the effect of both exercise interventions on VO_2_max and cardiometabolic factors, the uncertainty in effect will be expressed as 90% confidence limits and the likelihood the true value of the effect represents a substantial and clinically meaningful change (harm or benefit). Effects will be declared unclear if the confidence interval overlapped thresholds for substantiveness or the effect could be substantially positive and negative or beneficial and detrimental. All probabilistic magnitude-based inferences will be calculated using a published spreadsheet [27].

## Discussion

There has been a considerable amount of individual variability reported in the literature related to the response of CRF measurements (specifically, VO_2_max and peak aerobic ability [VO_2_peak]). However, there is still an overall lack of understanding as to why this variability occurs. Unfortunately, there is minimal consistency in methodology and the criteria for determining incidence of response leading to the overall findings indicating there are changes in training responsiveness of −33 to +76% [7]. However, some of the data associated with individual responses may be misleading as measurements were recorded as peak values [8, 28, 29] and may not be a direct representation of the maximal efforts for participants and, therefore, not an accurate representation of true physiological adaptations.

In order to have an all-inclusive definition for incidence of response the TE must be taken into account [10]. Therefore, it would be important to know biological variability and measurement error for each outcome to determine whether responses are beyond that of the TE. Two recent investigations [5, 30] utilized TE to determine response rate by defining a responder as an individual with improvements from pre- to post-training by > TE in a positive direction and, in contrast, an individual who improves by ≤ TE as a non-responder. Nevertheless, for the two aforementioned studies, values for day-to-day biological variability were used from previously published work and may not be directly applicable to the population being studied or the environmental conditions in which data collection takes place.

Conventionally, results of exercise-based studies are reported as the mean and standard deviation [31] and only illustrate the main effects and group differences of training responsiveness [32]. Overall, there is a lack in attention to individual differences with these conventional methods of reporting data since nearly 32% of measurements (distributed normally) fall outside of one standard deviation. Recent literature proposes reporting not only the mean, standard deviation, and group differences, but also individual responses to the training program [31, 32] or at least ranges of endurance changes [30]. This approach will strengthen study findings and provide further insight into the phenomenon of individual variability and training responsiveness.

From the HERITAGE Family Study [33], a large, well-controlled, 20-week standardized endurance training program, insight was gained on the incidence of response. It was reported that genetics may play a critical role in the incidence of response [34] with trainability of VO_2_max linked to familial aggregation [35]. However, a potentially overlooked factor in the individual variability may be linked to poor methodology of exercise prescription. Indeed, due to the theoretical and physiological mechanisms of exercise prescription, utilization of a threshold-based measurement for exercise prescription has been suggested to decrease the incidence of non-response and improve CRF and cardiometabolic factors compared to the traditional approach using intensities set relative to VO_2_max, HRmax, VO_2_R, or HRR [36]. However, there have been few studies that have reported individual responses following training relative to a threshold measurement [31]. To the best of our knowledge, there is currently only one study reporting individual responses to training comparing set intensities based on the first ventilatory threshold (VT1) and the second ventilatory threshold (VT2) measurements and percentage of HRR [5]. During incremental exercise, VT1 is the point at which increases in ventilation become non-linear (an increase in the ventilatory equivalents of oxygen [VE/VO_2_] with no increase in the ventilatory equivalents of carbon dioxide [VE/VCO_2_]) and VT2 is the point at which there is an accumulation of blood lactate due to the inability to buffer the amount of lactate produced (simultaneous increase in both VE/VO_2_ and VE/VCO_2_) [37].

Traditionally, exercise intensity has been prescribed based on a relative percent concept – based on a percentage of HRmax, VO_2_max, VO_2_R, or HRR. However, caution has been advised for utilization of the relative percent method, specifically HRmax and VO_2_max, as criteria to determine workload as they may not be sufficient to elicit the desired metabolic response [3, 38]. Furthermore, percentages for both HRmax and VO_2_max correspond to a wide range of exercise intensities relative to threshold measurements [39]. For example, with exercise intensities between 58% and 75% of VO_2_max, some participants were found to be above while others were reported to be below their individual anaerobic threshold [40]. Similar findings were noted when investigating a 12-month jogging/walking program [38]. In order to make the prescription of exercise intensity more individualized, many researchers have used percentages of HRR as this takes into consideration not only HRmax, but also resting HR. However, aerobic thresholds were found to be at 70% ± 10% of HRR [41] indicating large variability in the metabolic stress across individuals at a set percentage of HRR.

Indeed, genetics have gained a lot of attention to understand the specific roles of genes and response rates. However, based on the genetic evidence to date, there are many pathways that are associated with VO_2_max trainability and nearly an unlimited combination of signaling events that may influence the VO_2_max responsiveness [42, 43]. With genetics proposed to account for less than 50% of the variance in responsiveness, the other 50% is still not well understood.

One of the major areas in which the literature is lacking in the understanding of training responsiveness is the investigation of an individualized approach to exercise prescription and the time course changes. With the emerging concept of ‘exercise is medicine’ and the capacity to prescribe exercise to combat adverse effects of disease, the time course changes for VO_2_max and cardiometabolic risk factor measures need to be better understood to properly identify efficacious exercise doses (i.e., intensity, volume) that will elicit an adequate response. However, much of the literature on time course changes utilizes standardized methods of exercise prescription rather than individualized approaches. To the best of our knowledge, there is no literature in time course changes of VO_2_max and cardiometabolic risk factor outcomes with the use of a threshold-based protocol and exercise volume individualized based on kilocalories of expenditure per week with relation to body mass. Similarly, results of time course changes have traditionally been reported as only group means and standard deviations with individual time course changes not being reported. Based on a review of the literature, there have only been two studies to identify individual time course changes [44, 45]. Reporting of individual time course changes for VO_2_max and cardiometabolic risk factor measurements will help to further understand the individual variability in training responsiveness.

### Limitations

There are several limitations that merit discussion. It is possible there may be heterogeneity in training responses due to age alone given the large age range (30 to 75 years) that will be recruited for the current trial. However, the age range for the target sample will be comparable to previous studies [22, 23, 33] and also reflect the likely age range found in community exercise programs [46]. Another possible limitation is external validity given data collection will take place at moderate altitude. Nevertheless, to the best of our knowledge, there is no evidence to suggest differences in training responsiveness (i.e. responders and non-responders) between altitude-residing individuals and sea level counterparts. A third potential limitation is the inability to anticipate how many participants will be categorized as non-responders following the first 12-week intervention. Indeed, this limitation could arguably be the most significant due to the second part of the trial (crossing over non-responders to the other prescription group) being underpowered if there are a low number of non-responders. Nevertheless, to our knowledge, there are no previous investigations evaluating a crossover design to determine if non-responders can become responders with a different exercise prescription protocol. Therefore, even if the second part of the trial is underpowered, the results would provide valuable preliminary insight into the efficacy of individualized exercise prescription.

In summary, this original randomized controlled trial aims to (1) investigate the efficacy of an individualized exercise prescription at improving training responsiveness and, (2) to better understand the time course changes of training adaptations to both individualized and standardized exercise intensity prescription methods. It is anticipated that findings from this novel trial will add to our knowledge of how personalized exercise can enhance training efficacy and limit training unresponsiveness.

#### Study status

Recruitment will commence in September of 2016. The estimated completion date is mid-late 2018.

## Additional files

Additional file 1: SPIRIT checklist. (DOC 120 kb)
Additional file 2: Overall SPIRIT study schedule. (PDF 231 kb)

## Abbreviations

BP: blood pressure
CRF: cardiorespiratory fitness
CV: co-efficient of variation
GLM: general linear model
GXT: graded exercise test
HDL: high-density lipoprotein
HR: heart rate
HRR: heart rate reserve
ICC: intra-class correlation
IPAQ: International Physical Activity Questionnaire
kcal: kilocalorie
LDL: low-density lipoprotein
RHR: resting heart rate
SD: standard deviation
SPIRIT: Standard Protocol Items: Recommendations for Interventional Trials
TC: total cholesterol
TE: technical error
VE/VCO_2_: ventilatory equivalents of carbon dioxide
VE/VO_2_: ventilatory equivalents of oxygen
VO_2_max: maximal oxygen uptake
VO_2_peak: peak aerobic ability
VO_2_R: aerobic ability reserve
VT: ventilatory threshold
VT1: first ventilatory threshold
VT2: second ventilatory threshold
