# Supplementary material for: The incidence of training responsiveness to cardiorespiratory fitness and cardiometabolic measurements following individualized and standardized exercise prescription: study protocol for a randomized controlled trial
Source: Trials. 2016 Dec 19;17:601. doi: 10.1186/s13063-016-1735-0 (PMC5168814; doi:10.1186/s13063-016-1735-0)
Supplement: Additional file 2: — Overall SPIRIT study schedule. (PDF 231 kb) [file 13063_2016_1735_MOESM2_ESM.pdf]

Table 1 SPIRIT study calendar for the experimental group for the first 12 week intervention

			Exercise Intervention				
Point of time:	Pre-intervention	Baseline	Time 0	Week 4	Week 8	Week 12	12 Week Analysis
**Enrollment**							
Recruitment	x						
Medical history	x						
Inclusion criteria	x						
Randomization			x				
**Testing**							
Height		x		x	x	x	
Weight		x		x	x	x	
Resting heart rate		x		x	x	x	
Resting blood pressure		x		x	x	x	
Waist circumference		x		x	x	x	
Low-density lipoprotein		x		x	x	x	
High-density lipoprotein		x		x	x	x	
Triglycerides		x		x	x	x	
Blood glucose		x		x	x	x	
Maximal exercise test		x		x	x	x	
Verification test		x		x	x	x	
3-day nutrition recall		x				x	
**Analysis**							
Biological variability			x				
Time course changes							x
Incidence of response							x

Table 2 SPIRIT study calendar for the control group for the first 12 week intervention

Point of time:	Pre-intervention	Baseline	Time 0	Week 4	Week 8	Week 12	12 Week Analysis
**Enrollment**							
Recruitment	x						
Medical history	x						
Inclusion criteria	x						
**Testing**							
Height		x		x	x	x	
Weight		x		x	x	x	
Resting heart rate		x		x	x	x	
Resting blood pressure		x		x	x	x	
Waist circumference		x		x	x	x	
Low-density lipoprotein		x		x	x	x	
High-density lipoprotein		x		x	x	x	
Triglycerides		x		x	x	x	
Blood glucose		x		x	x	x	
Maximal exercise test		x		x	x	x	
Verification test		x		x	x	x	
3-day dietary recall		x				x	
IPAQ		x		x	x	x	
**Analysis**							
12 week changes							x

Table 3 SPIRIT study calendar (second 12 weeks) for VO_2max non-responders after the first 12 week intervention

			Exercise Intervention				
Point of time:	Pre-intervention	Baseline	Time 0	Week 4	Week 8	Week 12	12 Week Analysis
**Pre-Intervention**							
Switch of experimental group	X						
**Testing**							
Height		X		X	X	X	
Weight		X		X	X	X	
Resting heart rate		X		X	X	X	
Resting blood pressure		X		X	X	X	
Waist circumference		X		X	X	X	
Low-density lipoprotein		X					
High-density lipoprotein		X					
Triglycerides		X					
Blood glucose		X					
Maximal exercise test		X		X	X	X	
Verification test		X		X	X	X	
3-day nutrition recall		X				X	
**Analysis**							
Incidence of response							X
